# A Retropubic Cartilaginous Cyst in a Context of Surgery for Prostate Cancer

**DOI:** 10.5334/jbsr.2782

**Published:** 2022-04-27

**Authors:** Annelies Van Breda, Thibault Meert, Mathieu Lefere

**Affiliations:** 1 Department of Radiology, Imeldaziekenhuis Bonheiden, BE; 2 Department of Urology, Imeldaziekenhuis Bonheiden, BE

**Keywords:** retropubic cartilaginous cyst, suprapubic cartilaginous cyst, subpubic cartilaginous cyst, symphysis pubis, MRI, CT

## Abstract

**Teaching Point:** Retropubic cartilaginous cysts are rare, benign lesions originating from the symphysis pubis that should be considered in the differential diagnosis of a small slow-growing retropubic nodule.

## Case Presentation

**Figure 1:**
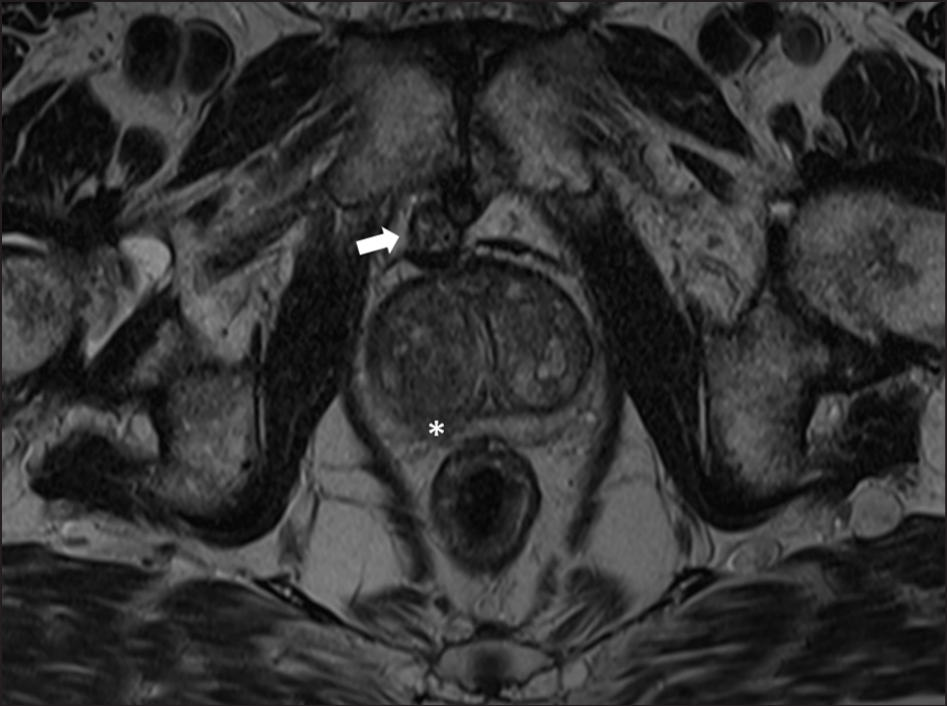


**Figure 2:**
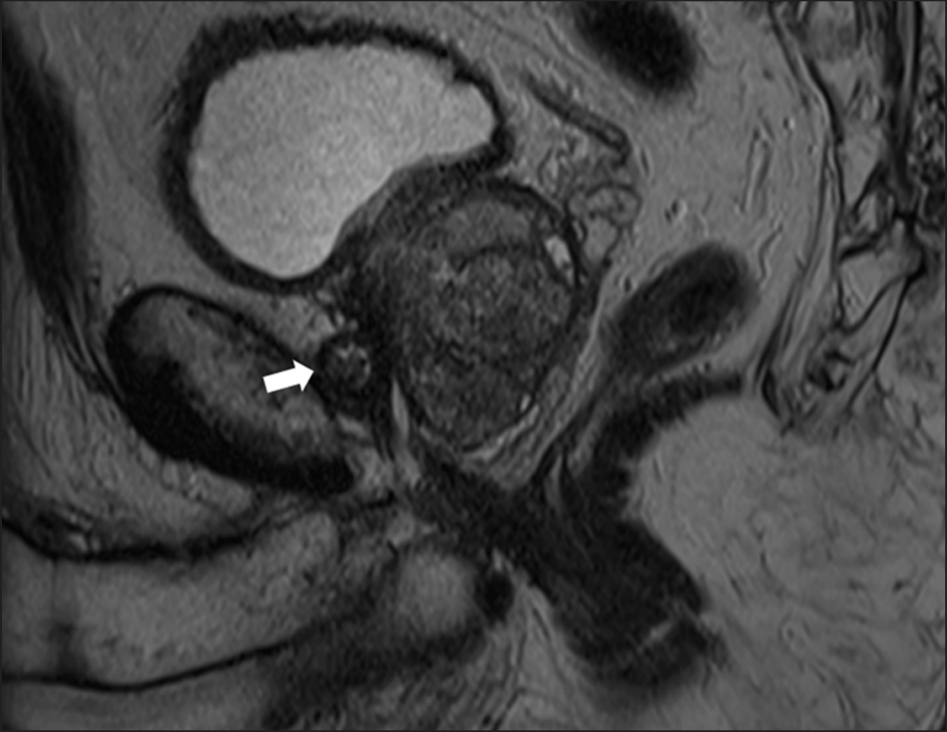


**Figure 3:**
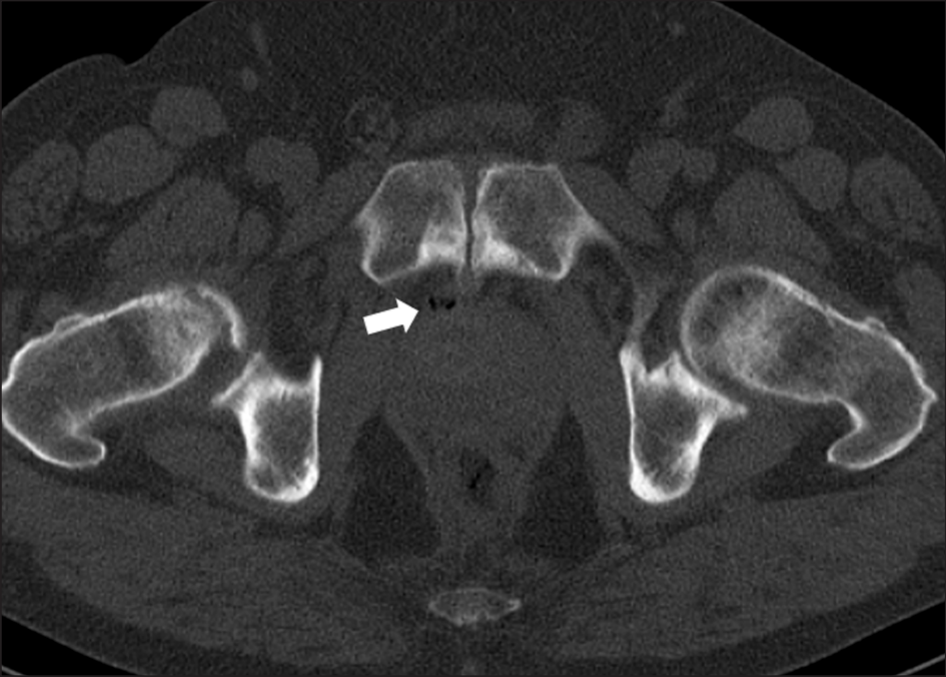


A 72-year-old man was referred to our department for a magnetic resonance imaging (MRI) scan of the prostate in the work-up of an elevated prostate-specific antigen (PSA). T2-weighted images (T2WI) showed a focal hypo-intense lesion in the right mid-portion of the peripheral zone of the prostate (***Figure 1***, asterisk) with slightly increased diffusion restriction, which was flagged as a possible malignancy. Subsequent transrectal biopsy and staging showed an intermediate risk prostate carcinoma cT2aN0M0, Gleason score 4+3, for which a robotic-assisted laparoscopic prostatectomy (RALP) was scheduled. During surgery, the urologist visualized a soft tissue nodule located posterior to the symphysis pubis, which had not been mentioned in the MRI-report. He immediately contacted the radiologist to re-assess the MRI scan in order to decide whether it should be resected. On MRI, a well-defined nodular structure measuring 17 mm was identified, lying immediately between the symphysis pubis and the prostate. It showed a heterogeneous intensity on axial (***Figure 1***, arrow) and sagittal (***Figure 2***, arrow) T2WI, and no diffusion restriction. There was clear growth when compared to a prostate MRI from one year prior. A recent single photon-emission computed tomography (SPECT-CT) scan showed degeneration of the symphysis, with the retropubic nodule containing small foci of gas (***Figure 3***, arrow). Because the nodule was identified as a benign retropubic cartilaginous cyst, unrelated to the prostate, it was left alone during the RALP procedure. Six months after surgery, the PSA level remained below 0.03 ng/ml.

## Comment

Retropubic cartilaginous cysts, also called supra- or subpubic cysts depending on their location relative to the symphysis, are rare cystic lesions originating from the symphysis pubis. Almost all reported cases concern multiparous and post-menopausal women with symptoms including urinary voiding difficulties, a painless slow-growing vulvar mass, or pelvic pain and dyspareunia [1]. Our case seems to be one of only very few reported cases in male patients. Ultrasound imaging may show a cystic structure adjacent to the symphysis. On MRI, the content is typically T1-hypo-intense and heterogeneous on T2WI, with a thin enhancing wall post-Gadolinium. Small foci of gas on CT can be very useful to confidently rule out a malignant soft-tissue mass. In our case, the heterogeneous T2 content closely resembled the hypertrophic nodules from the adjacent prostatic transition zone, creating a possible pitfall. Anatomopathological examination typically shows a collagenous capsule with fibrocartilaginous content and extensive mucinous degeneration. These cysts are believed to result from symphysis pubis degeneration. They are strictly benign but often show growth over time. Management should only be guided by patient’s symptoms [1]. Surgical resection can be considered in symptomatic patients, but the benefits are questionable, and potential complications include infection and symphysiolysis.
